# Comparison of Analgesic Efficacy of Ultrasound Guided PEC II Block Using Dexamethasone as an Adjuvant to Ropivacaine Versus Plain Ropivacaine in Patients Undergoing Modified Radical Mastectomy: A Double-Blind, Randomized Controlled Trial

**DOI:** 10.7759/cureus.58222

**Published:** 2024-04-14

**Authors:** Porika Prashanth Nayak, Sarita Ramchandani, Radhakrishna Ramchandani, Chandan Kumar Dey, Rashmi Dubey, Swati Vijapurkar

**Affiliations:** 1 Trauma and Emergency (Anesthesiology), All India Institute of Medical Sciences, Raipur, Raipur, IND; 2 Anesthesiology, All India Institute of Medical Sciences, Raipur, Raipur, IND; 3 General Surgery, All India Institute of Medical Sciences, Raipur, Raipur, IND

**Keywords:** modified radical mastectomy (mrm), opioid, pectoral nerve block, dexamethasone, ropivacaine

## Abstract

Introduction: Pain after breast cancer surgery is one of the main reasons for postoperative morbidity and pulmonary complications leading to increased hospital stay. Therefore, effective management of postoperative pain becomes necessary to alleviate patients' suffering and allow early mobilization and hospital discharge. Traditionally, opioids have been used to manage perioperative pain but they are associated with side effects. So, an opioid-sparing multimodal analgesia approach is used nowadays. Ultrasound-guided pectoral type-II (PEC II) block is increasingly being used to address acute postoperative pain after breast cancer surgery. However, to date, not many studies have been done regarding prolonging the duration of analgesia of PEC II blocks for postoperative pain relief in patients undergoing modified radical mastectomy (MRM). So, we undertook this study to compare the analgesic efficacy of PEC II block using dexamethasone as an adjuvant to ropivacaine versus plain ropivacaine in patients undergoing MRM.

Methods: After obtaining approval from the institute ethics committee and written informed consent from the patients, this prospective, double-blind, parallel group, randomized controlled study was carried out at All India Institute of Medical Sciences (AIIMS) Raipur, from March 2021 to March 2022. Sixty-four female patients, aged 18 years and above, belonging to the American Society of Anesthesiologists, physical status I, II, and III, undergoing unilateral, elective MRM under general anesthesia, were randomly allocated to two groups A and B, with 32 patients in each to receive 30 mL of 0.25% ropivacaine plus 2 mL (8 mg) of dexamethasone and 30 mL of 0.25% ropivacaine plus 2 mL of normal saline, respectively. The primary outcome measure was total opioid consumption till 12 hours postoperatively. Secondary outcome measures were the difference in pain scores based on the numeric rating scale till 12 hours postoperatively, post-operative sedation scores, the incidence of postoperative nausea vomiting (PONV), and other adverse events (if any).

Results: The mean (SD) of morphine (mg) consumed intraoperatively was 5.50 (1.05) and 5.95 (0.86) with P = 0.033 and that consumed postoperatively was 1.00 (0.00) and 1.69 (0.93) with P <0.001 in group A and B respectively, with morphine consumption being higher in the group. The difference in the NRS score for pain at rest was statistically significant at 2 h (*P*=0.030), 4 h (*P*=0.004), 6 h (*P*=0.002), and, 12 h (*P*=0.039) time points with the score being higher in group B. The groups were comparable in terms of postoperative sedation score (P > 0.05) and incidence of postoperative nausea and vomiting. None of the patients in group A and 6.2% of the patients in group B had nausea (P = 0.492). None of the patients in either of the groups had vomiting. No other complication occurred during the entire study in either of the groups.

Conclusion: In comparison to plain ropivacaine, the addition of dexamethasone as an adjuvant to ropivacaine for PEC II block in patients undergoing MRM significantly reduced perioperative opioid consumption and postoperative NRS scores. No significant change was noted in terms of postoperative sedation score, incidence of PONV, and other side effects between the groups. Therefore, we conclude that the analgesic efficacy of US-guided PEC II block using dexamethasone, as an adjuvant to ropivacaine is superior to that of plain ropivacaine in patients undergoing MRM.

## Introduction

Breast cancer is the most common cancer in women worldwide [1,2]. It accounts for about 25% of all cancers in women including Indian females [2,3]. Its management is multidisciplinary and involves a combination of radiotherapy, chemotherapy, and surgery. Surgery is the primary treatment modality [4] with modified radical mastectomy (MRM) being the gold standard surgical treatment. Pain after breast cancer surgery is one of the main reasons for postoperative morbidity and pulmonary complications leading to increased hospital stay [5]. Therefore, effectively managing postoperative pain receives utmost priority to alleviate patient's suffering, obtain early mobilization, reduce postoperative pulmonary complications, and provide early hospital discharge. Traditionally, opioids have been used to manage perioperative pain in breast cancer surgeries, but they are associated with side effects. So, an opioid-sparing, multimodal analgesia approach is used nowadays including basic analgesics like paracetamol and non-steroidal anti-inflammatory drugs, preoperative dexamethasone or gabapentin, regional analgesia techniques like paravertebral or pectoral nerves block and/or local anesthetic wound infiltration [6].

Due to its safety profile and ease of administration, the ultrasound (US)-guided pectoral type-II (PEC II) block has rapidly gained popularity in addressing acute postoperative pain after breast surgery [7]. The PEC block is an interfacial plane block between the pectoralis major and minor muscles, first described by Blanco et al. in 2011 [8]. It later became to be known as the PEC I block [7]. Later in 2012, Blanco et al. introduced the PEC II block or modified PEC block [9]. However, to date, not many studies have been done regarding prolonging the duration of analgesia of PEC II blocks for postoperative pain relief in patients undergoing MRM. So, we undertook this study to compare the analgesic efficacy of PEC II block using dexamethasone as an adjuvant to ropivacaine versus plain ropivacaine in patients undergoing MRM.

## Materials and methods

The procedures followed in this study were as per the ethical standards of the Institute Ethics Committee (IEC) and the Declaration of Helsinki, 2013. After obtaining approval from the IEC (vide letter number 1362/IEC-AIIMSRPE/2020, dated: 13/11/2020), the study was registered prospectively in the clinical trials registry ‑ India (vide registration number CTRI/2021/03/031781, dated: March 8, 2021). Written informed consent was obtained from all the patients before enrolling them to study. The study was carried out at All India Institute of Medical Sciences (AIIMS) Raipur, which is a tertiary care center, from March 2021 to March 2022.

In this prospective, double-blind, parallel-group, randomized controlled study, 64 female patients, aged 18 years and above, belonging to the American Society of Anesthesiologists (ASA), physical status I, II, and III, scheduled to undergo unilateral, elective MRM under general anesthesia (GA), were included. Patients who refused to give consent, with body mass index (BMI) ≥ 35 kg/m^2^ or body weight ≤ 40 kg, having contraindications to regional anesthesia, allergy to study drugs, chest wall deformity, having undergone prior breast surgery, suffering from psychiatric illness, and pregnant/lactating women and patients who suffered from any untoward major intraoperative complication like severe hypotension, bradycardia, neuraxial injury (if any) were excluded from this study. The primary outcome measure of this study was total opioid (morphine) consumption till the 12-hour (h) time point post-operatively. Secondary outcome measures were the difference in pain scores based on the numeric rating scale (NRS) in the first 12 hours period post-operatively, post-operative sedation scores, incidence of postoperative nausea vomiting (PONV), and adverse events (if any).

Using a computer-generated randomization sequence, patients were randomly allocated to two groups, A and B with 32 patients in each. Group allocation was concealed in a sealed, opaque envelope which was randomly picked up by the patient in the pre-operative area and was handed over to a qualified healthcare worker (HCW) who was not part of the study. Then after opening the envelope, the HCW prepared the study drugs as per the group allocation and handed them over to the anesthesiologist performing the block. Thus, all the persons involved in the study including the patients, the anesthesiologist performing the block, and the person collecting the data and providing post-operative care were blinded to group allocation. The Consolidated Standards of Reporting Trials (CONSORT) guidelines were followed during the conduct of this study. The details of the study were explained to the patients one day before the surgery.

On receiving the patients in the operation room (OR), standard ASA monitors were applied and baseline values of the patient's vital parameters including systolic blood pressure (SBP), diastolic blood pressure (DBP), oxygen saturation of arterial blood (SpO_2_), and heart rate (HR) were recorded. The standard institutional protocol was followed for administration of GA and all patients were induced with intravenous (IV) propofol 2 mg/kg and IV morphine 0.1 mg/kg. Vecuronium 0.1mg/kg IV was used to facilitate the insertion of the endotracheal tube. Anesthesia was maintained with isoflurane and oxygen: nitrous oxide mixture in a 50:50 ratio. After securing the airway, under all aseptic precautions, US guided PEC II block was administered using a high-frequency (6-13 MHz) linear probe. As per the group allocation, group A patients received 30 mL of 0.25% ropivacaine along with 2 mL of dexamethasone (8 mg) whereas group B patients received 30 mL of 0.25% ropivacaine along with 2mL of normal saline (NS). Study drugs were injected at two different sites. The first injection, containing 10 mL of the drug was injected between the pectoral major and minor muscles while the second injection, containing the remaining 20 mL volume of the drug was injected between the pectoralis minor and serratus anterior muscle. Thereafter the surgeons were allowed to proceed with surgery. During surgery, analgesia and muscle relaxation were maintained with intermittent boluses of morphine at the rate of 0.05 mg/kg and vecuronium at the rate of 0.015 mg/kg, respectively, to keep HR and BP within 20% of the baseline values. Continuous hemodynamic monitoring was done, and the patient's vital parameters were recorded at every 10-minute (min) interval till the end of surgery. Intraoperative complications (if any) were recorded and managed as per the standard institutional protocol. After completion of surgery, reversal of the neuromuscular blockade was done with IV neostigmine 0.05 mg/kg and IV glycopyrrolate 0.01 mg/kg. After extubation, patients were transferred to the post-anesthesia care unit (PACU), and ASA standard monitors were applied. The time of arrival of the patients in the PACU was considered as zero (0) h and patient-controlled analgesia (PCA) was commenced with the administration of IV morphine 1 mg as a bolus, with a lock-out interval of 10 min and four-hour limit of 0.25 mg/kg without any baseline infusion. Postoperative adverse events (if any) were recorded and treated as per the standard protocol followed at our institute.

Postoperatively, the patients were assessed for pain at rest and sedation, by an investigator blinded to the group allocation. The pain was assessed using NRS with a score of “zero (0)” considered no pain and “ten (10)” considered the worst imaginable pain. Sedation was assessed using Modified Ramsay’s eight-pointed sedation score [10] with “one (1)” being completely awake and “eight (8)” being unresponsive to external stimuli. Observations were recorded at zero, two, four, six, and 12 h time points postoperatively. The total amount of morphine consumed in the first 12 hours after surgery was noted. The patients were not administered any analgesic other than morphine in the postoperative period. At the end of the study, patients were asked to rate their satisfaction score with pain management (Patient Satisfaction Score) on a 5-point scale (5-Excellent, 4-Very good, 3-Good, 2-Fair, 1-Poor).

The data were recorded in a Microsoft (MS) Excel spreadsheet version 365 (Microsoft Corporation, Redmond, WA, USA), and analysis was done using statistical software SPSS version 21 (IBM Corp., Armonk, NY, USA). Continuous and categorical variables were expressed as mean (SD) and percentage (%), respectively. Student’s t-tests were applied to compare the two groups. Repeated measures - analysis of variance (RM-ANOVA) was used to compare changes in parameters at each time interval. Statistical significance was kept at P < 0.05.

The sample size was calculated based on a previous study done by Kaur et al. [11], which showed 20 mg as the minimum baseline requirement of morphine. It was decided to take at least a 10% decrease in the requirement of morphine to consider it clinically significant. Therefore, an estimate of 18 mg of morphine was taken for this study. The dispersion of the values was set on point estimate ± 2.7. The alpha and beta values for the study were traditionally set at 1.96 and 0.84, respectively, and sample size calculation was done using the formula,

n=2×(𝑍𝛼+𝑍𝛽)2×σ2𝛿2

where,

n = sample size, 𝑍𝛼 =1.96 at 95% confidence interval, 𝑍𝛽 = 0.84 at 80% power, σ = Combined SD (2.7), 𝛿 = difference between mean (2).

Substituting the values, the sample size came out to be 29 in each group. Estimating 10% dropouts, an additional three cases were added in each group thus making a total sample size of 64 with 32 patients per group.

## Results

**Figure 1:**
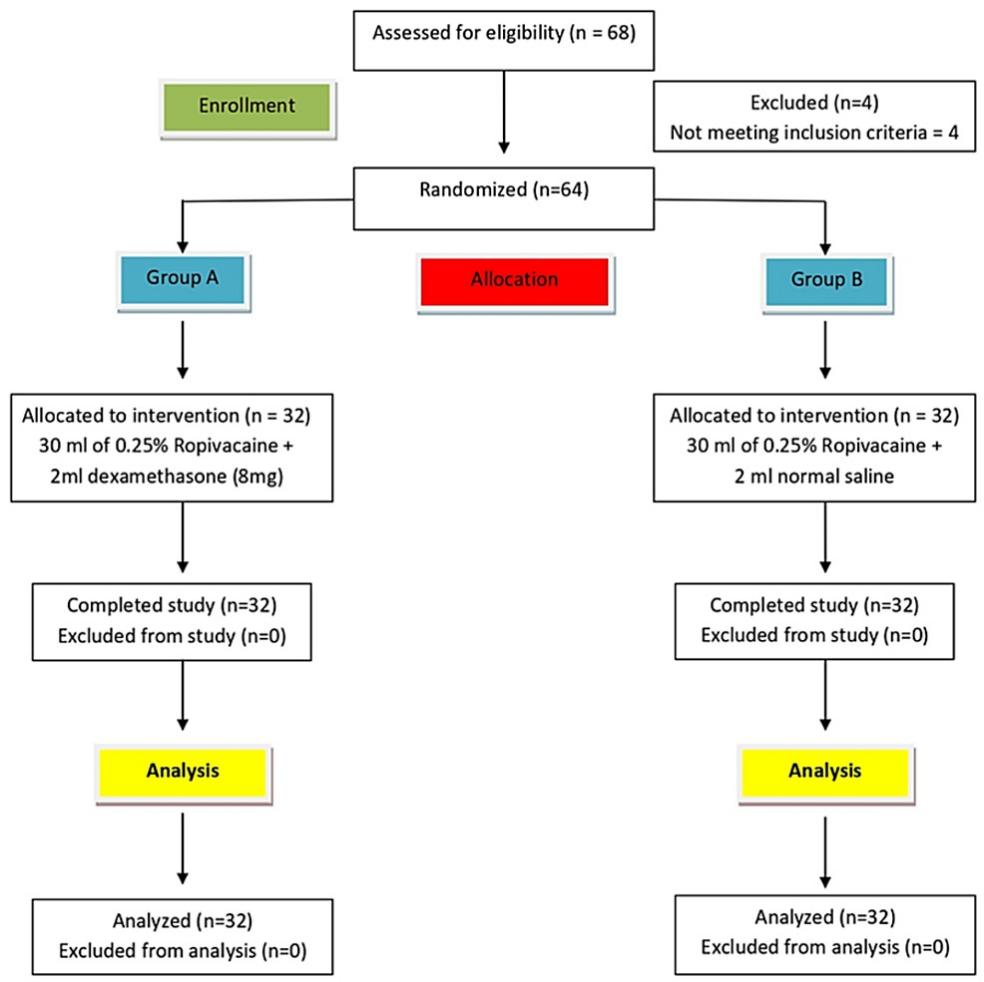
Consolidated Standards of Reporting Trials (CONSORT) flow chart

**Figure 2:**
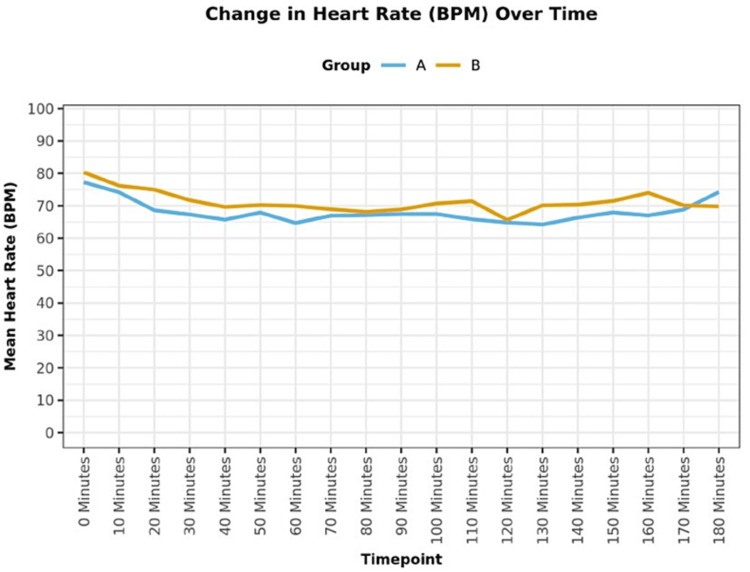
Line diagram showing the change in the intraoperative heart rate over time between the two groups BPM = beats per minute.

**Figure 3:**
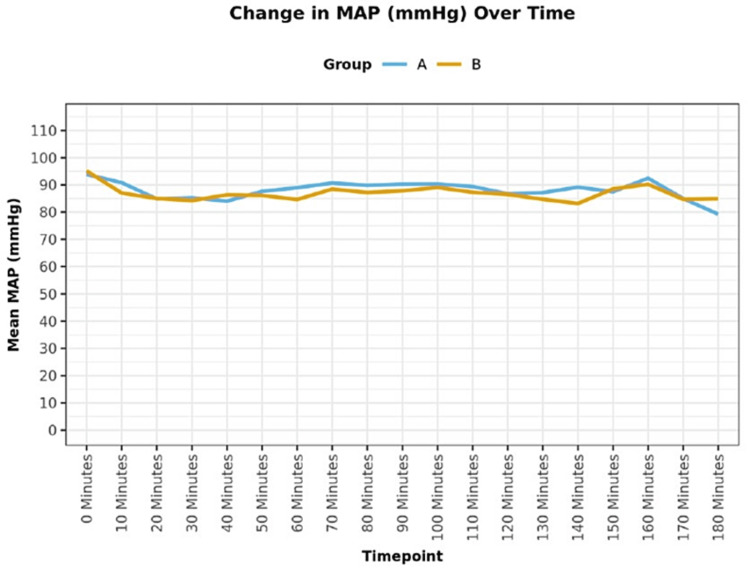
Line diagram showing the change in intraoperative MAP (mmHg) over time between the groups MAP = Mean arterial pressure.

Sixty-four patients received the allocated intervention and completed the final analysis (Figure 1). The groups were comparable in terms of demographic characteristics (Table 1). On comparing the groups, in terms of HR (bpm) over time intraoperatively, a statistically significant difference was found between the groups (P = 0.036) with mean HR being higher in group B as compared to group A (Figure 2). The groups were comparable in terms of MAP (mmHg) over time during the intraoperative period (P = 0.616; Figure 3). The groups were comparable in terms of duration of surgery. The mean (SD) of the duration of surgery (minutes) was 145 (27.70) and 154.68 (38.77) in groups A and B, respectively, with P = 0.577 (Table 1). The mean (SD) of morphine (mg) consumed intraoperatively was 5.50 (1.05) and 5.95 (0.86) with a median interquartile range (IQR) of 5.5 (4.5-6) and 6 (5-6.5) and a range of 4-8 and 4.5-8 in groups A and B, respectively, with the intraoperative morphine consumption being higher in group B (P = 0.033; Table 1). The mean (SD) of morphine (mg) consumed in the first 12 hours postoperatively was 1.00 (0.00) and 1.69 (0.93) with a median (IQR) of 1 (1-1) and 1 (1-2.25) with a range of 1-1 and 1-4 in groups A and B, respectively, with the postoperative morphine consumption being higher in group B (P < 0.001; Table 1). The groups were comparable in terms of intraoperative hypotension and bradycardia. Around 3.1% of the participants in group A and none (0.0%) of the participants in group B had intraoperative hypotension (P = 1.000; Table 1). None (0.0%) of the participants in either of the groups had intraoperative bradycardia (Table 1). On comparison of groups, in terms of NRS score for pain at rest, a statistically significant difference was found at 2 h (P = 0.030), 4 h (P = 0.004), 6 h (P = 0.002), and 12 h (P = 0.039) time points postoperatively, with NRS score being higher in group B (Table 2). The groups were comparable in terms of postoperative sedation scores (P > 0.05; Table 3), incidence of PONV (Table 1), and patient satisfaction scores (Table 1). None (0.0%) of the patients in group A and 6.2% of the patients in group B had nausea (P = 0.492; Table 1). None of the patients in either of the groups had vomiting (Table 1). The mean (SD) of the patient satisfaction score was 4.38 (0.61) and 4.12 (0.75) with a median (IQR) of 4 (4-5) and 4 (4-5) and a range of 3-5 and 2-5 in groups A and B, respectively, and the difference was not statistically significant (P = 0.192; Table 1). No complication occurred during the entire study in either of the groups.

**Table 1:** Demographic and clinical parameters Data are presented as mean (SD), and number (percentage). BMI = Body mass index, n = number, % = percentage. P-value < 0.05 is considered statistically significant. 1: t-test, 2: Wilcoxon-Mann-Whitney U Test, 3: Fisher's Exact Test

	Group		
Parameters	A (n = 32)	B (n = 32)	P-value
Age (Years)	53.00 (10.09)	50.03 (12.40)	0.298^1^
Weight (kg)	55.44 (9.66)	57.64 (8.70)	0.341^1^
BMI (kg/m²)	21.98 (3.80)	23.14 (3.47)	0.127^2^
Hypotension (Yes)	1 (3.1%)	0 (0.0%)	1.000^3^
Duration of Surgery (minutes)	145 (27.70)	154.68 (38.77)	0.577^2^
Intra-Operative Morphine Consumption (mg)	5.50 (1.05)	5.95 (0.86)	0.033^2^
Post-Operative Morphine Consumption (mg)	1.00 (0.00)	1.69 (0.93)	< 0.001^2^
Nausea (Yes)	0 (0.0%)	2 (6.2%)	0.492^3^
Patient Satisfaction Score	4.38 (0.61%)	4.12 (0.75%)	0.192^2^

**Table 2:** Post-operative NRS score over various time points Data are presented as mean (SD). NRS=Numeric Rating Scale, n=number. P-value < 0.05 is considered statistically significant. 1: Wilcoxon-Mann-Whitney U Test.

Time points (h)	NRS Score		P-value
	Group A (n = 32)	Group B (n = 32)	
0	0.00 (0.00)	0.22 (0.79)	0.082^1^
2	0.19 (0.40)	0.62 (0.87)	0.030^1^
4	0.84 (0.68)	1.50 (0.98)	0.004^1^
6	1.28 (0.63)	1.78 (0.61)	0.002^1^
12	1.56 (0.56)	1.84 (0.45)	0.039^1^

**Table 3:** Post-operative sedation score over various time points Data are presented as mean (SD). n=number. P-value < 0.05 is considered statistically significant. 1: Wilcoxon-Mann-Whitney U Test.

Time points (h)	Post-operative Sedation Score		P-value
	Group A (n = 32)	Group B (n = 32)	
0	2.09 (0.47)	1.88 (0.66)	0.067^1^
2	1.06 (0.25)	1.16 (0.37)	0.238^1^
4	1.00 (0.00)	1.00 (0.00)	-
6	1.00 (0.00)	1.00 (0.00)	-
12	1.00 (0.00)	1.00 (0.00)	-

## Discussion

The present study aimed to evaluate the analgesic efficacy of US-guided PEC II block using dexamethasone as an adjuvant to ropivacaine vs plain ropivacaine in patients undergoing MRM. In our study, both groups were comparable in terms of demographic characteristics (P > 0.05). Intra-operatively, the mean HR was found to be higher in group B than in group A (P = 0.036) and the difference was statistically significant. There was no significant difference in the change in MAP (mmHg) over time between the two groups (P = 0.616). Both groups were comparable with respect to the duration of surgery (P = 0.577). The intraoperative and postoperative morphine consumption was more in group B as compared to group A and the difference was statistically significant (P < 0.05). These findings of our study were similar to the findings of Parrington et al. [12], Vieira et al. [13], and Carolyne et al. [14]. Parrington et al. [12] in their study to determine whether the addition of dexamethasone to mepivacaine prolongs the duration of analgesia after supraclavicular brachial plexus block, found that the fentanyl requirements in PACU were significantly reduced in dexamethasone group as compared to control group (P =0.020). Similarly, Vieira et al. [13] in their study to determine whether the addition of dexamethasone to interscalene brachial plexus block prolongs the duration of sensory analgesia, noted that the 24 h opioid requirement was lower in the dexamethasone group (0 mg, IQR 0-10 mg vs. 30 mg, IQR 13-50 mg; P < 0.0001) as compared to the control group. Carolyne et al. [14] in their systematic review to evaluate the comparative efficacy and safety of perineural dexamethasone versus placebo for postoperative pain control, found that the cumulative 24-h postoperative opioid consumption was significantly lower in the perineural dexamethasone group as compared to the placebo group.

Postoperatively, no significant difference was found in the NRS scores between the groups at zero-hour time points, but a statistically significant difference was found at two-hour, four-hour, six-hour, and twelve-hour time points. The maximum difference was noted at a six-hour time point with the overall NRS score being higher in group B. These findings were similar to those of Parrington et al. [12], Carolyne et al. [14], and Elisabeth et al. [11]. Parrington et al. [12] noted significantly reduced VAS pain scores eight hours after surgery in the dexamethasone group as compared to the control group (P =0.005). Carolyne et al. [14] reported that the postoperative pain intensity at 12 and 24 h time points was significantly lower in the perineural dexamethasone group as compared to the control group. Similar results were found in the study conducted by Elisabeth et al. [15] in which the authors reported a lower VAS score at an 11 h time point postoperatively in the dexamethasone group compared to the placebo group.

In our study, the groups were comparable in terms of postoperative sedation scores over various time points and the difference was not found to be statistically significant. The safety profile of the blocks in the two groups was assessed in terms of the incidence of side effects and the difference was not found to be statistically significant (P > 0.05) In our study, the groups were comparable in terms of patient satisfaction score which was assessed at 12 h time point postoperatively, showing no statistically significant difference between the groups (P= 0.192). These findings were similar to the findings of Vieira et al. [13] in which the authors noted no significant difference in the patient satisfaction score at 48 h time point postoperatively (9.5, IQR 8-10 vs. 8.0, IQR 8-10; dexamethasone vs. control, respectively).

Limitation

There were a few limitations to our study. Firstly, we compared the analgesic efficacy of PEC II block using dexamethasone as an adjuvant to ropivacaine versus plain ropivacaine therefore the results of our study cannot be generalized to other forms of regional analgesia techniques or the systemic administration of dexamethasone. Secondly, this study was limited to a homogenous population of female breast cancer patients aged 18 years or above, scheduled for elective unilateral MRM so the results may not be applied to other patient populations comprising pediatric patients or male breast cancer. Thirdly, the results of our study are based on the data from a single tertiary care center. Results may have been different in the case of a multi-centric study.

## Conclusions

In our study, we found that in comparison to plain ropivacaine, the addition of dexamethasone as an adjuvant to ropivacaine for PEC II block in patients undergoing MRM significantly reduced intra and post-operative opioid consumption. It also significantly reduced NRS scores at various time points postoperatively. No significant change was noted in terms of postoperative sedation score, the incidence of PONV, or other side effects between the two groups. Therefore, we conclude that the analgesic efficacy of US-guided PEC II block using dexamethasone, as an adjuvant to ropivacaine is superior to that of plain ropivacaine in patients undergoing MRM.
